# Characterizing populations prioritized for PrEP in 19 African countries: a review of national guidance

**DOI:** 10.1002/jia2.26407

**Published:** 2025-01-05

**Authors:** Lauren A. Graybill, Caroline N. McKay, Jiayu Wang, Nadia A. Sam‐Agudu, Marcel Yotebieng, Friday Saidi, Linda‐Gail Bekker, Bonnie E. Shook‐Sa, Benjamin H. Chi, Nora E. Rosenberg

**Affiliations:** ^1^ Institute for Global Health and Infectious Diseases University of North Carolina at Chapel Hill Chapel Hill North Carolina USA; ^2^ Department of Health Behavior University of North Carolina at Chapel Hill Chapel Hill North Carolina USA; ^3^ International Research Center of Excellence Institute of Human Virology Nigeria Abuja Nigeria; ^4^ Department of Paediatrics and Child Health University of Cape Coast School of Medical Sciences Cape Coast Ghana; ^5^ Department of Pediatrics Global Pediatrics Program and Division of Infectious Diseases University of Minnesota Medical School Minneapolis Minnesota USA; ^6^ Division of General Internal Medicine Department of Medicine Albert Einstein College of Medicine Bronx New York USA; ^7^ UNC Project Malawi Lilongwe Malawi; ^8^ Department of Obstetrics and Gynecology University of North Carolina at Chapel Hill Chapel Hill North Carolina USA; ^9^ Desmond Tutu HIV Centre University of Cape Town Cape Town South Africa; ^10^ Department of Biostatistics University of North Carolina at Chapel Hill Chapel Hill North Carolina USA

**Keywords:** HIV prevention, pre‐exposure prophylaxis, policy review, priority populations, Africa, operational and implementation sciences

## Abstract

**Introduction:**

While African countries have expanded access to HIV pre‐exposure prophylaxis (PrEP) since 2015, regional targets for PrEP uptake remain unmet. Understanding which populations are prioritized for PrEP at the policy level is an important step in determining the scope of PrEP distribution across Africa and identifying gaps in programme implementation. We reviewed national guidance to characterize populations prioritized for PrEP in Africa.

**Methods:**

Between January and June 2023, we searched for current National HIV Treatment and Prevention Guidelines, National HIV Strategic Plans, and the United States President's Emergency Plan for AIDS Relief (PEPFAR) Country Operational Plans (COPs) for all African countries implementing PrEP programmes supported by PEPFAR in 2022. From each document, we summarize the populations prioritized for PrEP within a country and describe PrEP eligibility.

**Results:**

In 2022, 19 African countries implemented PrEP programmes supported by PEPFAR. Eighteen of these countries contributed National Guidelines (2016−2022), 18 contributed National Strategic Plans (2017−2023) and 19 contributed COPs (2022) to this review. Twenty‐nine population groups were prioritized for PrEP in these documents. All countries prioritized HIV‐serodifferent couples, female sex workers (FSWs), adolescent girls and young women (AGYW), pregnant and breastfeeding women (PBFW) and people who inject drugs (PWID), and most prioritized men who have sex with men (MSM; *n* = 18), transgender people (*n* = 18) and people in prisons (*n* = 17). The remaining 21 populations were prioritized in fewer than two‐thirds of countries.

**Discussion:**

FSWs, MSM, PWID, transgender people and people in prisons were typically prioritized for PrEP with no eligibility restrictions. In contrast, most countries had at least one document indicating that HIV‐serodifferent couples, AGYW and PBFW were only eligible for PrEP if classified as high risk. Few documents specified how risk was determined, and no document included validated HIV risk assessment tools to guide implementation. We observed similarities in priority populations across countries with different HIV epidemics and inconsistencies in who was prioritized for PrEP within a country's own set of policy documents.

**Conclusions:**

Understanding how PrEP prioritization policies impact HIV incidence in different epidemiologic settings is critical for strengthening PrEP implementation across the continent.

## 1 INTRODUCTION

Despite substantial progress in HIV testing and treatment across Africa, progress in primary HIV prevention remains inadequate. In 2022, an estimated 660,000 new HIV acquisitions occurred in east, south and west‐central Africa, accounting for 51% of the total number of new HIV acquisitions globally [1]. This number must be radically reduced to meet the Joint United Nations Programme on HIV/AIDS (UNAIDS) target of fewer than 200,000 annual new HIV acquisitions globally by 2030 [2].

Daily oral HIV pre‐exposure prophylaxis (PrEP) is a highly effective biomedical intervention for HIV prevention. While African countries—especially those in southern and eastern Africa—have progressively scaled up PrEP since 2015 [3, 4], regional targets for PrEP uptake remain unmet [5]. Suboptimal uptake of PrEP reflects a complex array of barriers at individual, interpersonal and structural levels. At the structural level, national policies governing how PrEP is prioritized play a critical role in shaping the availability and accessibility of PrEP within a country. Notably, global frameworks guiding the development of these policies have differed across institutions and time.

In 2012, the World Health Organization (WHO) initially recommended PrEP for specific population groups: female sex workers (FSWs), men who have sex with men (MSM), people who inject drugs (PWID), transgender people, people in prisons and other closed settings, and HIV‐serodifferent couples [6, 7]. Then, in 2015, the WHO endorsed PrEP for any population with substantial HIV risk, which was defined as an HIV incidence rate >3 per 100 person‐years (PY) in the absence of PrEP [8]. In 2022, WHO guidance was updated to recommend a client‐centred approach that considers individual choice, local contextual factors, individual behaviours and partner characteristics when determining who may benefit from PrEP [9]. In contrast, early guidance from UNAIDS recommended targeted PrEP delivery to priority populations in geographic areas at elevated risk and acknowledged that priority populations would likely differ across epidemics [10]. While newer UNAIDS guidance continues to emphasize the importance of tailored PrEP implementation according to geographic and population considerations, it also highlights the role of individual and partner characteristics in determining HIV risk [11].

In light of global guidance that has evolved and, at times, presented conflicting priorities, we conducted a review of national PrEP guidance to characterize the populations prioritized for PrEP across Africa. By systematically identifying prioritized populations for PrEP in national policies, our work provides a comprehensive overview of the current landscape and offers insights into one of the key structural determinants of PrEP uptake. Such evidence is essential to guide future efforts aimed at optimizing PrEP delivery and accelerating progress towards global HIV prevention targets.

## 2 METHODS

### 2.1 Document selection

We restricted our analysis to the 19 African countries implementing a PrEP programme funded by the United States President's Emergency Plan for AIDS Relief (PEPFAR) in 2022: Botswana, Burundi, Cameroon, Côte d'Ivoire, Democratic Republic of the Congo (DRC), Eswatini, Ethiopia, Kenya, Lesotho, Malawi, Mozambique, Namibia, Nigeria, Rwanda, South Africa, Tanzania, Uganda, Zambia and Zimbabwe. For each country, we obtained publicly available National HIV Treatment and Prevention Guidelines, National Strategic Plans and PEPFAR Country Operational Plans (COPs). These documents were selected for this review because they offer different insights on PrEP implementation in each country.

National Guidelines provide detailed, evidence‐based recommendations on clinical care for the diagnosis, treatment and prevention of HIV. These technical documents are primarily aimed at healthcare providers and ensure standardized and effective care for individuals living with or at risk of HIV. In contrast, National Strategic Plans are comprehensive policy documents that describe a country's broader strategy for addressing the HIV epidemic. They often outline a multisectoral approach to mobilizing resources, coordinating activities and integrating services designed to reach ambitious HIV treatment and prevention goals. Finally, COPs summarize HIV treatment and prevention activities that are guided by a major donor's priorities.

Between January and July 2023, we searched for National Guidelines and National Strategic Plans on the Ministry of Health websites, the PrEPWatch website (www.prepwatch.org) and the Differentiated Service Delivery website (www.differentiatedservicedelivery.org). All COPs were downloaded from the U.S. Department of State's website (www.state.gov). When more than one version of a country's National Guideline, National Strategic Plan or COP was available, we downloaded the most recently published version. No document was excluded based on language; all non‐English documents (*n* = 5 in French, *n* = 2 in Portuguese) were translated using Google Document Translate (https://translate.google.com/).

### 2.2 Data abstraction

For each document, we recorded the document type, title, date published and information on the population(s) prioritized for PrEP in a standardized data abstraction tool in Microsoft Excel. Populations prioritized for PrEP were not predefined; they were identified as those (1) explicitly targeted for PrEP, (2) described as eligible for PrEP or (3) reported to already be receiving PrEP in each policy document. For each priority population, we recorded all population‐specific eligibility criteria that would restrict that population's access to PrEP. In instances where a particular population's PrEP eligibility differed within the same document, we reported the more specific criterion.

Two reviewers (CNM and JW) abstracted data from at least two of each document type to reach a consensus on coding. One reviewer (CNM) then abstracted data from the remainder of documents, flagging those with ambiguous or conflicting information. A third reviewer (LAG) reviewed abstracted data from these flagged documents. All disagreements were resolved through discussion.

### 2.3 Data analysis

We describe population groups prioritized for PrEP by country and document type. First, to provide a comprehensive overview of priority populations in national guidance, we summarized the population groups prioritized for PrEP within each country's set of documents. For this analysis, we used a country's set of documents as a single source of information guiding PrEP implementation for that country. Thus, if a population was prioritized for PrEP in any of a country's documents, we classified the population as a priority for PrEP in that country. Then, to better understand consistency in guidance within a country's set of documents, we stratified according to country and document type and summarized which documents prioritized each population group and the circumstances under which each priority population was eligible for PrEP. For this analysis, we classified a population as fully eligible if the document did not specify any restrictions, and partly eligible if the document specified that only certain members of a prioritized population were eligible for PrEP. Given the number of priority populations identified in national guidance, we restricted this part of the analysis to the population groups prioritized for PrEP in at least two‐thirds of countries.

## 3 RESULTS

In total, 18 National Guidelines, 18 National Strategic Plans and 19 COPs were included in our review (Table 1 and Appendix S1). The 18 National Guidelines reflected clinical guidelines for HIV service delivery starting in 2016 (*n* = 3), 2019–2020 (*n* = 7), and 2021–2022 (*n* = 8), while the 18 National Strategic Plans summarized the overall national HIV response strategy for an average span of 5 years, starting in 2017–2018 (*n* = 8), 2019–2020 (*n* = 4), and 2021–2023 (*n* = 6). We were unable to locate National Guidelines for Côte d'Ivoire or the National Strategic Plan for Burundi. All COPs reflected PEPFAR‐supported activities planned for 2023. In general, COPs provided the most current guidance (all were published in 2022), followed by National Guidelines (average publication year: 2020) and National Strategic Plans (average publication year: 2019).

**Table 1:** Summary of PrEP policy documents.

	National Guidelines		National Strategic Plans		Country Operational Plans	
	Publication year	Title	Publication year	Title	Publication year	Title
**Eastern Africa (*n* = 6 countries)**						
**Burundi**	2020	Directives Nationales Pour la Prevention et le Traitement du VIH au Burundi (*National Guidelines for the Prevention and Treatment of HIV in Burundi*)	*Not found*		2022	Burundi Country Operational Plan (COP) 2022 Strategic Direction Summary
**Ethiopia**	2022	National Guidelines for Comprehensive HIV Prevention, Care, and Treatment: Pocket Guide 2022	*Not reported*	HIV/AIDS National Strategic Plan for Ethiopia 2021–2025	2022	Ethiopia Country Operational Plan (COP) 2022 Strategic Direction Summary
**Kenya**	2022	Kenya HIV Prevention and Treatment Guidelines 2022	*Not reported*	Kenya AIDS Strategic Framework II 2020/21−2024/25	2022	Kenya Country Operational Plan 2022 Strategic Direction Summary
**Rwanda**	2020	National Guidelines for Prevention and Management of HIV, *Edition 2020*	2018	Rwanda HIV and AIDS National Strategic Plan 2013–2018: Extension 2018–2020	2022	Rwanda Country Operational Plan (COP/ROP) 2022 Strategic Direction Summary
**Tanzania**	2019	National Guidelines for the Management of HIV and AIDS, *7th Edition*	2017	Health Sector HIV and AIDS Strategic Plan 2017–2022	2022	Tanzania Country Operational Plan COP 2022 Strategic Direction Summary
**Uganda**	2020	Consolidated Guidelines for Prevention and Treatment of HIV and AIDS in Uganda, 2020	2020	National HIV and AIDS Strategic Plan 2020/2021−2024/2025	2022	Uganda Country Operational Plan (COP) 2022 Strategic Direction Summary
**Southern Africa (*n* = 9 countries)**						
**Botswana**	2016	Handbook of the Botswana 2016 Integrated HIV Clinical Care Guidelines	2019	The Third Botswana National Strategic Framework for HIV and AIDS 2019–2023	2022	Botswana Country Operational Plan (COP/ROP) 2022 Strategic Direction Summary
**Eswatini**	2022	Eswatini Integrated HIV Management Guidelines 2022	2018	The National Multisectoral HIV and AIDS Strategic Framework (NSF) 2018–2023	2022	Eswatini Country Operational Plan (COP) 2022 Strategic Direction Summary
**Lesotho**	2022	National Guidelines on the Use of Antiretroviral Therapy for HIV Prevention and Treatment *Sixth Edition*	2018	National HIV & AIDS Strategic Plan 2018/19–2022/23	2022	Lesotho Country Operational Plan (COP 2022) Strategic Direction Summary
**Malawi**	2020	National Guidelines for the Provision of Oral Pre‐Exposure Prophylaxis for Individuals at Substantial Risk of HIV in Malawi	2020	Malawi National Strategic Plan for HIV and AIDS 2020–2025	2022	Malawi Country Operational Plan 2022 Strategic Direction Summary
**Mozambique**	2021	Guião de Oferta da Profilaxia Pré‐Exposição ao HIV (*Guide to Delivering HIV Pre‐Exposure Prophylaxis*)	2021	Plano Estratégico Nacional de Combate ao HIV e SIDA (PEN V), 2021–2025 (*National Strategic Plan of the Response to HIV and AIDS, 2021–2025*)	2022	Country Operational Plan (COP 2022) Strategic Direction Summary PEPFAR Mozambique
**Namibia**	2021	National Guidelines for Antiretroviral Therapy, Pocket Guide 2021	2017	National Strategic Framework for HIV and AIDS Response in Namibia 2017/18 to 2021/22	2022	Namibia Country Operational Plan 2022 Strategic Direction Summary
**South Africa**	2020	Guidelines for the Provision of Pre‐Exposure Prophylaxis (PrEP) to Persons at Substantial Risk of HIV Infection	2023	National Strategic Plan for HIV, TB and STIs, 2023–2028	2022	Country Operational Plan PEPFAR South Africa 2022 Strategic Direction Summary
**Zambia**	2022	Zambia Consolidated Guidelines for Treatment and Prevention of HIV Infection	2017	National HIV and AIDS Strategic Framework 2017–2021	2022	Zambia Country Operational Plan (COP) 2022 Strategic Direction Summary
**Zimbabwe**	2016	Guidelines for Antiretroviral Therapy for the Prevention and Treatment of HIV in Zimbabwe	2020	Zimbabwe National AIDS Strategic Plan III 2021–2025	2022	Zimbabwe Country Operational Plan (COP) 2022
**West‐central Africa (*n* = 4 countries)**						
**Cameroon**	2021	Directives Nationales sur la Prise en Charge du VIH (*National HIV Care Guidelines)*	*Not reported*	Cameroon National Strategic Plan for fight against HIV/AIDS and STIs 2021–2023	2022	Cameroon Country Operational Plan COP 2022 Strategic Direction Summary
**Côte d'Ivoire**	*Not found*		2020	Plan Stratégique National de lutte contre le VIH, le SIDA, et les infections sexuellement transmissibles 2021–2025 (*National Strategic Plan for the fight against HIV, AIDS and sexually transmitted infections 2021–2025*)	2022	Côte d'Ivoire Country Operational Plan (COP/ROP) 2022 Strategic Direction Summary
**Democratic Republic of Congo**	2016	Guide de Prise en Charge Integree du VIH en Republique Democratique du Congo (*Guide to Integrated HIV Care in the Democratic Republic of Congo*)	2018	Plan Stratégique National de La Riposte au VIH/SIDA 2018–2021 (*National Strategic Plan for the Response to HIV/AIDS 2018–2021*)	2022	Democratic Republic of the Congo Country Operational Plan (COP) 2022 Strategic Direction Summary
**Nigeria**	2020	National Guidelines for HIV Prevention, Treatment and Care	*Not reported*	National HIV and AIDS Strategic Plan 2017–2021	2022	Nigeria Country Operational Plan (COP) 2022 Strategic Direction Summary

Consistent with WHO and UNAIDS guidance from 2015 onwards [8, 10], all policy documents—except National Strategic Plans for Tanzania (2017) and the DRC (2018)—recommended PrEP as an additional HIV prevention strategy for people/populations at high risk of acquiring HIV. From these documents, 29 populations, largely defined according to individual and partner characteristics, behaviours, and vulnerabilities, were identified as a priority for PrEP (Table 2). Countries prioritized an average of 11 populations for PrEP, with national guidance from Ethiopia prioritizing the fewest populations (*n* = 5), while national guidance from South Africa prioritizing the most (*n* = 14). On average, countries located in southern Africa prioritized more populations for PrEP than countries in eastern or west‐central Africa (*n* = 12, 10 and 9, respectively).

**Table 2:** Population groups prioritized for PrEP within national guidance.

	Eastern Africa						Southern Africa									West‐central Africa				Number of countries with any guidance prioritizing the population for PrEP	
	Burundi	Ethiopia	Kenya	Rwanda	Tanzania	Uganda	Botswana	Eswatini	Lesotho	Malawi	Mozambique	Namibia	South Africa	Zambia	Zimbabwe	Cameroon	Cote d'Ivoire	DRC	Nigeria	*n*	(%)
HIV‐serodifferent couples	Yes	Yes	Yes	Yes	Yes	Yes	Yes	Yes	Yes	Yes	Yes	Yes	Yes	Yes	Yes	Yes	Yes	Yes	Yes	19	(100%)
Female sex workers	Yes	Yes	Yes	Yes	Yes	Yes	Yes	Yes	Yes	Yes	Yes	Yes	Yes	Yes	Yes	Yes	Yes	Yes	Yes	19	(100%)
Adolescent girls and young women	Yes	Yes	Yes	Yes	Yes	Yes	Yes	Yes	Yes	Yes	Yes	Yes	Yes	Yes	Yes	Yes	Yes	Yes	Yes	19	(100%)
Pregnant and breastfeeding women	Yes	Yes	Yes	Yes	Yes	Yes	Yes	Yes	Yes	Yes	Yes	Yes	Yes	Yes	Yes	Yes	Yes	Yes	Yes	19	(100%)
People who inject drugs	Yes	Yes	Yes	Yes	Yes	Yes	Yes	Yes	Yes	Yes	Yes	Yes	Yes	Yes	Yes	Yes	Yes	Yes	Yes	19	(100%)
Men who have sex with men	Yes	No	Yes	Yes	Yes	Yes	Yes	Yes	Yes	Yes	Yes	Yes	Yes	Yes	Yes	Yes	Yes	Yes	Yes	18	(95%)
Transgender people	Yes	No	Yes	Yes	Yes	Yes	Yes	Yes	Yes	Yes	Yes	Yes	Yes	Yes	Yes	Yes	Yes	Yes	Yes	18	(95%)
People in prison	No	No	Yes	Yes	Yes	Yes	Yes	Yes	Yes	Yes	Yes	Yes	Yes	Yes	Yes	Yes	Yes	Yes	Yes	17	(89%)
People with multiple sexual partners	No	No	Yes	No	Yes	Yes	Yes	Yes	Yes	No	Yes	Yes	Yes	Yes	Yes	Yes	No	No	No	12	(63%)
People with STIs	No	No	Yes	No	Yes	Yes	No	Yes	Yes	Yes	Yes	Yes	Yes	Yes	Yes	Yes	No	No	No	12	(63%)
People with inconsistent condom use	No	No	Yes	No	Yes	Yes	Yes	Yes	Yes	No	Yes	No	Yes	Yes	Yes	Yes	No	No	No	11	(58%)
Men classified as “high‐risk”	No	No	Yes	No	Yes	No	No	Yes	Yes	No	Yes	Yes	No	Yes	Yes	No	Yes	Yes	No	10	(53%)
People who use recreational drugs or alcohol	No	No	Yes	No	No	Yes	Yes	Yes	No	No	No	Yes	Yes	No	Yes	Yes	No	No	No	8	(42%)
People with a partner(s) of unknown status	No	No	Yes	No	No	Yes	No	Yes	Yes	No	Yes	No	Yes	Yes	Yes	No	No	No	No	8	(42%)
Men who engage in transactional sex	No	No	Yes	Yes	No	Yes	Yes	No	Yes	Yes	Yes	No	No	No	No	No	No	No	No	7	(37%)
People with recurrent PEP	No	No	Yes	No	Yes	Yes	No	No	No	No	Yes	Yes	No	Yes	Yes	No	No	No	No	7	(37%)
People who request PrEP	No	No	No	No	No	No	No	Yes	Yes	No	No	Yes	Yes	Yes	Yes	No	Yes	No	No	7	(37%)
People who are migrant/mobile	No	No	No	Yes	No	Yes	No	No	Yes	No	No	Yes	No	Yes	Yes	No	No	No	No	6	(32%)
Young men	No	No	No	No	No	No	No	Yes	No	No	No	No	No	Yes	No	No	No	No	Yes	3	(16%)
People in “high‐risk” occupations (not sex work)	No	No	No	No	No	Yes	No	No	No	No	Yes	No	No	Yes	No	No	No	No	No	3	(16%)
People experiencing violence or abuse	Yes	No	No	No	No	No	No	No	No	No	No	Yes	No	No	Yes	No	No	No	No	3	(16%)
People who identify as part of the LGBTQ community	No	No	No	No	No	No	No	No	No	No	No	No	Yes	No	No	No	No	No	No	1	(5%)
Men with young female partners	No	No	No	No	No	No	Yes	No	No	No	No	No	No	No	No	No	No	No	No	1	(5%)
Men in high‐incidence geographies	No	No	Yes	No	No	No	No	No	No	No	No	No	No	No	No	No	No	No	No	1	(5%)
People with disabilities	No	No	No	No	No	No	No	No	No	No	No	No	Yes	No	No	No	No	No	No	1	(5%)
People with mental health conditions	No	No	No	No	No	No	No	No	No	No	No	No	Yes	No	No	No	No	No	No	1	(5%)
Orphans	No	No	No	No	No	No	No	No	No	No	No	No	Yes	No	No	No	No	No	No	1	(5%)
Homeless children	No	No	No	No	No	No	No	No	No	No	No	No	Yes	No	No	No	No	No	No	1	(5%)
People who live in rural areas, informal settlements and inner cities	No	No	No	No	No	No	No	No	No	No	No	No	Yes	No	No	No	No	No	No	1	(5%)
**Number of populations prioritized for PrEP**	8	5	13	10	9	13	11	12	12	9	12	12	14	13	13	9	9	9	9		

Abbreviations: LGBTQ, lesbian, gay, bisexual, transgender and queer or questioning; PEP, post‐exposure prophylaxis; STI, sexually transmitted infection.

PrEP policies largely reflected early WHO recommendations on offering PrEP to FSWs, MSM, PWID, transgender people, people in prisons and HIV‐serodiscordant couples [6, 7]. All 19 countries in this review prioritized FSWs, PWID and HIV‐serodifferent couples for PrEP, and most countries also prioritized MSM (*n* = 18), transgender people (*n* = 18) and people in prisons (*n* = 17). Notable exceptions included Ethiopia, which did not have policies that prioritized MSM, transgender people, or people in prisons for PrEP, and Burundi, which did not have policies that prioritized people in prisons.

More recent guidance from the WHO and UNAIDS has emphasized the importance of individual choice, local contextual factors, and individual and partner characteristics when determining who would benefit from PrEP [9−11]. While all 19 countries prioritized AGYW and pregnant and breastfeeding women (PBFW) for PrEP, fewer than two‐thirds of countries prioritized population groups defined by specific factors associated with HIV risk, such as people with multiple sex partners (*n* = 12), people with a history of sexually transmitted infections (STIs; *n* = 12), people with a history of inconsistent condom use (*n* = 11), people with partners of unknown HIV status (*n* = 8) and people who request PrEP (*n* = 7). Notably, countries in southern Africa were considerably more likely to prioritize these population groups for PrEP than countries in eastern and west‐central Africa.

Among the eight population groups prioritized for PrEP in at least two‐thirds of countries (HIV‐serodifferent couples, FSWs, AGYW, PBFW, PWID, MSM, transgender people and people in prisons), we observed variability in which policy documents prioritized each of the population groups for PrEP and the circumstances under which each priority population was eligible for PrEP (Table 3). Variability was related to the period between the oldest and newest documents in a country. Countries with shorter periods (1−2 years) provided inconsistent guidance for an average of three population groups, whereas countries with longer periods of 3–4 years and 5–6 years provided inconsistent guidance for an average of four and five population groups, respectively.

**Table 3:** Summary of national guidance on population groups frequently prioritized for PrEP in Africa.

	HIV‐serodifferent couples			Female sex workers			Adolescent girls and young women			Pregnant and breastfeeding women			People who inject drugs			Men who have sex with men			Transgender people			People in prisons		
	National Guidelines	National Strategic Plans	Country Operational Plans	National Guidelines	National Strategic Plans	Country Operational Plans	National Guidelines	National Strategic Plans	Country Operational Plans	National Guidelines	National Strategic Plans	Country Operational Plans	National Guidelines	National Strategic Plans	Country Operational Plans	National Guidelines	National Strategic Plans	Country Operational Plans	National Guidelines	National Strategic Plans	Country Operational Plans	National Guidelines	National Strategic Plans	Country Operational Plans
**Eastern Africa**																								
Burundi	Yes	NA	Yes*	NS	NA	Yes	NS	NA	Yes*	NS	NA	Yes*	NS	NA	Yes	NS	NA	Yes	NS	NA	Yes	NS	NA	NS
Ethiopia	Yes	Yes*	Yes	Yes	Yes	Yes	NS	NS	Yes^a^	Yes*	NS	Yes*	NS	NS	Yes^a^	NS	NS	NS	NS	NS	NS	NS	NS	NS
Kenya	Yes*	Yes	Yes	Yes	Yes	Yes	NS	Yes	Yes	Yes	NS	Yes*	Yes*	Yes	Yes	NS	Yes	Yes	NS	Yes	Yes	NS	Yes	Yes
Rwanda	Yes*	Yes	Yes*	Yes*	Yes	Yes*	NS	Yes	Yes*	Yes*	NS	Yes*	NS	Yes	NS	Yes*	Yes	Yes*	NS	Yes	Yes*	NS	Yes	NS
Tanzania	Yes*	NS	NS	Yes	NS	Yes	Yes*	NS	Yes*	Yes*	NS	Yes	Yes	NS	Yes	Yes	NS	Yes	Yes	NS	Yes	NS	NS	Yes
Uganda	Yes	Yes	Yes	Yes	Yes	Yes	Yes	Yes*	Yes	Yes*	NS	Yes	Yes	Yes	Yes	Yes	Yes	Yes	Yes*	Yes	Yes	Yes*	Yes	Yes
**Southern Africa**																								
Botswana	Yes	NS	NS	Yes	Yes	Yes	NS	Yes	Yes*	NS	NS	Yes	Yes	NS	Yes	Yes	Yes	Yes	NS	NS	Yes	NS	NS	Yes
Eswatini	Yes	Yes	Yes	Yes	Yes	Yes	Yes	Yes	Yes	Yes	Yes	Yes	Yes*	NS	Yes	Yes	Yes	Yes	Yes	NS	Yes	NS	NS	Yes
Lesotho	Yes*	Yes	Yes	Yes	Yes	Yes	NS	Yes*	Yes*	Yes	Yes	Yes*	Yes	NS	Yes*	Yes	Yes	Yes	Yes*	Yes	Yes*	Yes	Yes	Yes
Malawi	Yes*	Yes	Yes	Yes	Yes	Yes	Yes*	Yes	Yes	Yes*	Yes	Yes*	NS	Yes	NS	Yes	Yes	Yes	Yes	Yes	Yes	NS	NS	Yes
Mozambique	Yes*	Yes	Yes	Yes	Yes	Yes	Yes*	Yes*	Yes	Yes*	Yes*	Yes	Yes	Yes	Yes	Yes	Yes	Yes	Yes	Yes	Yes	Yes	Yes	Yes
Namibia	Yes*	Yes	Yes	NS	Yes	Yes	NS	Yes	Yes	Yes*	Yes	Yes	Yes	Yes	NS	NS	Yes	Yes	NS	NS	Yes	NS	Yes	NS
South Africa	Yes*	Yes*	NS	Yes	Yes	Yes	Yes	Yes*	Yes*	Yes*	NS	Yes*	Yes	Yes	Yes	Yes	Yes	Yes	NS	Yes	Yes	NS	Yes	Yes
Zambia	Yes*	Yes	Yes	Yes	Yes	Yes	Yes	Yes	Yes	Yes*	NS	Yes	Yes	Yes	Yes	Yes	Yes	Yes	Yes	Yes	Yes	Yes	Yes	Yes
Zimbabwe	Yes*	Yes	Yes	Yes	Yes	Yes	Yes	Yes*	Yes	Yes*	Yes*	Yes	Yes*	Yes	NS	Yes	Yes	Yes	Yes	Yes	Yes	Yes	NS	NS
**West‐central Africa**																								
Cameroon	NS	NS	Yes^a^	Yes*	Yes	Yes*	NS	NS	Yes^a^	NS	NS	Yes^a^	NS	Yes	Yes^a^	Yes	Yes	Yes*	NS	Yes	Yes^a^	NS	NS	Yes^a^
Côte d'Ivoire	NA	NS	Yes	NA	Yes	Yes	NA	NS	Yes	NA	NS	NS^b^	NA	Yes	NS	NA	Yes	Yes	NA	Yes	Yes	NA	Yes	NS
DRC	Yes	NS	Yes*	Yes	NS	Yes	Yes*	NS	Yes	NS	NS	Yes	Yes	NS	NS	Yes	NS	Yes	Yes	NS	Yes	NS	NS	Yes
Nigeria	Yes	NS	Yes*	Yes	Yes	Yes	Yes	NS	Yes	Yes	NS	Yes*	Yes	Yes	Yes	Yes	Yes	Yes	Yes	NS	Yes	Yes	NS	Yes

*Note*: NA = policy document was not available for review; NS = population was not specified as a priority for PrEP in the policy document; Yes = population was prioritized for PrEP in the policy document with no restrictions on eligibility specified; Yes* = population was prioritized for PrEP in the policy document with restrictions on eligibility.

^a^ Policy document indicated the country was in the process of formally adding this population to a list of priority populations for PrEP.

^b^ Policy document outlines efforts to advocate for the inclusion of this group as a priority population.

### 3.1 HIV‐serodifferent couples

The WHO has recommended PrEP for HIV‐serodifferent couples since 2012 [6]. All countries had at least one policy document specifying that HIV‐negative partners in HIV‐serodifferent relationships were a priority for PrEP. This population was prioritized for PrEP in nearly all National Guidelines (*n* = 17/18) and in most COPs (*n* = 16/19). They were less frequently specified as a priority population in National Strategic Plans (*n* = 12/18). All documents from Uganda and Eswatini classified HIV‐negative people in serodifferent relationships as a priority population with no eligibility criteria specified. Documents from 10 other countries also consistently prioritized this population for PrEP, though most of these countries had at least one document—typically their National Guideline—specifying that the HIV‐negative partner was eligible for PrEP only if their partner living with HIV was not on antiretroviral therapy (ART) or on ART without viral suppression. Policy documents from the seven remaining countries provided inconsistent guidance.

### 3.2 Female sex workers

The WHO has recommended PrEP for FSWs since 2014 [7]. All countries had at least one policy document specifying that FSWs were a priority for PrEP. This population was prioritized for PrEP in nearly all National Guidelines (*n* = 16/18) and National Strategic Plans (*n* = 16/18), and in all COPs (*n* = 19/19). Documents from 13 countries consistently classified FSWs as a priority population with no eligibility criteria specified. All documents from Rwanda and Cameroon also prioritized FSWs for PrEP; however, eligibility criteria varied within each of these country's guidelines. While National Strategic Plans from both countries specified no eligibility criteria, other guidance indicated that FSWs were eligible for PrEP if they were aged 18+ (Cameroon, COP), aged 21+ (Cameroon, National Guidelines) or engaging in condomless sex (Rwanda, National Guidelines and COP). FSWs were specified as a priority population for PrEP in only certain documents from Burundi (COP only), the DRC (National Guideline and COP), Namibia (National Strategic Plan and COP) and Tanzania (National Guideline and COP).

### 3.3 Adolescent girls and young women

While no guidance from the WHO has explicitly included AGYW as a priority population for PrEP, UNAIDS has emphasized this population group as a priority for HIV prevention in southern and eastern Africa since 2015 [10]. All countries had at least one policy document specifying that AGYW were a priority for PrEP, though countries defined AGYW differently with age ranges from 10 to 29 years old. While this population was only prioritized for PrEP in approximately 50% of National Guidelines (*n* = 10/18) and two‐thirds of National Strategic Plans (*n* = 12/18), they were included as a priority population in all COPs (*n* = 19/19). AGYW were consistently prioritized for PrEP in documents from seven countries (all in eastern and southern Africa) and inconsistently prioritized for PrEP in documents from the remaining 12 countries. National Strategic Plans from all countries in west‐central Africa, and National Guidelines from most countries in eastern Africa, did not specify whether AGYW were a priority population for PrEP. For three countries—Burundi, Ethiopia and Cameroon—the only document that included AGYW as a priority population was the COP.

In most countries, at least one policy document indicated that AGYW were eligible for PrEP only under limited circumstances. The most common eligibility criterion was being “high‐risk,” though the definition of risk was rarely provided. When defined, elements of risk included: a current or prior pregnancy, having a partner living with HIV or a partner of unknown HIV status, age‐disparate relationships, inconsistent condom use, multiple sex partners, transactional sex, STIs, prior use of post‐exposure prophylaxis and intimate partner violence. National guidance typically used a combination of these elements to define risk—and combinations were unique to each document type reviewed.

### 3.4 Pregnant and breastfeeding women

The WHO has recommended PrEP for PBFW with high HIV risk since 2017 [12]. All countries had at least one policy document specifying that PBFW were a priority for PrEP. This population was prioritized for PrEP in most National Guidelines (*n* = 14/18) and nearly all COPs (*n* = 18/19). They were less frequently specified as a priority population in National Strategic Plans (*n* = 6/18). All policy documents from six African countries (all in southern Africa) prioritized PBFW for PrEP, though only one of these countries had national guidance that consistently prioritized PBFW for PrEP with no eligibility criteria specified (Eswatini). Documents from the remaining 13 countries provided conflicting guidance on whether PBFW were a priority population for PrEP. PBFW were not prioritized for PrEP in National Strategic Plans for any country in eastern and west‐central Africa. Notably, for two countries—Burundi and Cameroon—the only document that included PBFW as a priority population was the COP.

Most documents that prioritized PBFW indicated this population was only eligible for PrEP under limited circumstances. The most common eligibility criterion was being “high‐risk,” which was often undefined. When defined, common elements of risk included: having a partner living with HIV or a partner of unknown HIV status, having a partner with multiple partners, having a partner who belongs to a key population, having a partner who has a high‐risk occupation, perceiving oneself to be at‐risk of HIV, belonging to a key population group, history of STIs or history of transactional sex. National guidance frequently used a combination of these elements to define risk, and combinations were unique to each document type reviewed.

### 3.5 People who inject drugs

The WHO has recommended PrEP for PWID since 2014 [7]. All countries had at least one policy document specifying that PWID were a priority for PrEP. This population was prioritized for PrEP in most National Guidelines (*n* = 13/18), National Strategic Plans (*n* = 12/18) and COPs (*n* = 13/19). Documents from Mozambique, Nigeria, South Africa, Uganda and Zambia consistently included PWID as a priority population with no eligibility criteria specified. PWID were also consistently prioritized for PrEP in documents from Kenya, though PrEP eligibility differed across documents. While no eligibility criteria were specified in the Kenyan COP or National Strategic Plan, Kenyan National Guidelines indicated that PWID were eligible for PrEP if they shared needles and syringes. Documents from the remaining 13 countries varied with respect to whether PWID were prioritized and the circumstances within which PWID were eligible for PrEP. Condomless sex and sharing injection equipment were common eligibility criteria. Notably, for two countries—Burundi and Ethiopia—the only document that included PWID as a priority population was the COP.

### 3.6 Men who have sex with men

The WHO has recommended PrEP for MSM since 2012 [6]. Most countries (*n* = 18/19) had at least one policy document specifying that MSM were a priority for PrEP. This population was prioritized for PrEP in most National Guidelines (*n* = 14/18) and National Strategic Plans (*n* = 15/18), and in almost all COPs (*n* = 18/19). Documents from 11 countries consistently identified MSM as a population eligible for PrEP with no eligibility criteria specified. All documents from Cameroon and Rwanda also prioritized MSM for PrEP, but MSM were eligible under differing circumstances within each country's documents. In Cameroon, for example, the COP indicated that only MSM aged 18+ were eligible, while the National Strategic Plan and National Guidelines did not specify any eligibility criteria. MSM were specified as a priority population for PrEP in only certain documents from Burundi (COP only), the DRC (National Guidelines and COP), Kenya (National Strategic Plan and COP), Namibia (National Strategic Plan and COP) and Tanzania (National Guidelines and COP).

### 3.7 Transgender people

The WHO has recommended PrEP for transgender people since 2012 [6]. Most countries (*n* = 18/19) had at least one policy document specifying that transgender people were a priority for PrEP. While this population was only prioritized for PrEP in just over half of National Guidelines (*n* = 10/18) and National Strategic Plans (*n* = 11/18), they were included as a priority population in almost all COPs (*n* = 18/19). The COP was the only document that specified transgender people as a priority population for Burundi and Botswana.

Policy documents from Côte d'Ivoire, Malawi, Mozambique, Zambia and Zimbabwe consistently classified transgender people as eligible for PrEP with no eligibility criteria specified. All documents from Lesotho and Uganda also prioritized transgender people, though PrEP eligibility varied within each country's guidelines. While National Strategic Plans for both countries—and the Ugandan COP—indicated no eligibility criteria for transgender people, other policy documents indicated that transgender people were eligible for PrEP if they were in high‐incidence geographies (Lesotho, COP), a transgender woman (Lesotho, National Guidelines) or engaging in condomless sex (Uganda, National Guidelines). Documents from 10 countries provided inconsistent guidance on whether transgender people were a priority population for PrEP and—when included as a priority population—whether they were eligible under all or limited circumstances.

### 3.8 People in prisons

The WHO has recommended PrEP for people in prisons and other closed settings since 2014 [7]. Most countries (*n* = 17/19) had at least one policy document specifying that people in prisons were a priority for PrEP. Notably, this population was prioritized for PrEP in only one‐third of National Guidelines (*n* = 6/18) and one‐half of National Strategic Plans (*n* = 9/18). They were more commonly prioritized in COPs (*n* = 13/19). Notably, the COP was the only document that prioritized people in prison for PrEP in Botswana, Cameroon, the DRC, Eswatini, Malawi and Tanzania. Lesotho, Mozambique and Zambia were the only countries with documents that consistently included people in prisons as a priority population for PrEP. When prioritized, most documents indicated this population was eligible for PrEP with no eligibility criteria specified.

## 4 DISCUSSION

In this review of National HIV Treatment and Prevention Guidelines, National Strategic Plans and COPs, we characterized the population groups prioritized for PrEP across 19 African countries and contextualized findings within global guidance from the WHO and UNAIDS. Twenty‐nine population groups, largely defined according to individual and partner characteristics, behaviours and vulnerabilities, were prioritized for PrEP in included countries. All countries prioritized HIV‐serodifferent couples, FSWs, AGYW, PBFW and PWID for PrEP, and most countries also prioritized MSM, transgender people and people in prisons for PrEP. In each country's set of documents, there was variability in which documents prioritized each of these population groups for PrEP and whether these population groups were eligible for PrEP under all or limited circumstances. Twenty‐one other priority populations, largely defined according to specific factors associated with HIV risk, were identified in a small subset of countries. We observed regional differences in the prioritization of these population groups.

National PrEP policies broadly reflected early WHO guidance, which recommended offering PrEP to FSWs, MSM, PWID, transgender people and people in prisons [6, 7]. Because these vulnerable populations comprise a small portion of the total population in Africa [13], and account for a large proportion of new HIV acquisitions in many African countries [14], prioritizing these groups for PrEP is important. Laws criminalizing sex work, same‐sex sexual acts or relationships and injection drug use, however, are pervasive across Africa and sabotage efforts towards epidemic control [15]. HIV prevalence among MSM, for example, is five times greater in countries with laws criminalizing same‐sex relationships compared to those without, and 12 times greater in countries with recent prosecutions for same‐sex relationships than in those without [16]. There is also evidence that repressive policing of sex work increases the risk of HIV among FSWs [17], and that the criminalization of drug use negatively impacts HIV treatment and prevention efforts among PWID [18]. Thus, while national policies in Africa largely reflect the critical need for HIV prevention in these populations, their impact on PrEP utilization may be limited without efforts to decriminalize key populations across Africa.

HIV‐serodifferent couples, AGYW and PBFW were also frequently identified as priority populations for PrEP in Africa. Notably, these groups were typically eligible for PrEP only under limited circumstances associated with increased HIV risk. While a risk‐stratified approach to PrEP offer is reasonable—these groups comprise a large proportion of the total population in Africa and HIV risk in each group varies according to individual‐, partner‐ and community‐level factors—validated HIV risk assessment tools developed for HIV‐serodifferent couples [19], AGYW [20] and PBFW [21] in Africa were notably absent from all National Guidelines, National Strategic Plans and COPs. Instead, countries relied on unvalidated screening tools for determining PrEP eligibility, which may be insufficient for measuring HIV risk [22]. Future studies are needed to evaluate whether implemented strategies are effective for triaging patients according to HIV risk, particularly as more effective—and expensive—PrEP modalities become more widely available in Africa.

Several population groups at increased HIV risk were inconsistently prioritized for PrEP across Africa. While STIs, inconsistent condom use, multiple sex partners and having a partner of unknown HIV status are established predictors of HIV acquisition in Africa [23−25], these population groups were prioritized for PrEP in fewer than one‐half of countries in eastern and western/central Africa. People who request PrEP were also infrequently prioritized for PrEP in national guidance in eastern and western/central Africa, despite explicit guidance from the WHO on the importance of prioritizing this population for PrEP [6]. These oversights highlight an important gap between guidance from the WHO and UNAIDS, which emphasizes how HIV risk is shaped by the combination of local HIV transmission potential and individual and partner‐level behaviours and characteristics [6, 25], and national guidance, which often failed to consider broader contextual and behavioural attributes of HIV risk when determining who may benefit from PrEP services.

We observed similarities in the populations prioritized for PrEP across African countries with different HIV epidemics. For example, AGYW were prioritized for PrEP in all countries included in this review despite relatively low HIV incidence among AGYW in eastern and west/central Africa (0.2 and 0.1 per 100PY, respectively) compared to southern Africa (1.3 per 100PY) [26]. Population‐based approaches to PrEP prioritization are practical and align with global guidance, but they often overlook critical variations in local HIV transmission dynamics. Analyses using nationally representative data from 15 African countries, for example, demonstrate that population viraemia is the strongest predictor of HIV incidence among men and women [27, 28]. These findings highlight the potential to refine PrEP prioritization by integrating local epidemiologic data, such as subnational population viraemia, into decision‐making frameworks. Future research is needed to evaluate how such approaches could complement population‐based strategies to better allocate PrEP to those at greatest risk.

We observed inconsistencies in who was prioritized for PrEP within a country's own set of policy documents. Discrepancies included differences in whether a population was prioritized, whether there were population‐specific eligibility criteria, and what those criteria were. Differences across documents are not, necessarily, contradictory policies. They may reflect unintentional omissions or changes over time. Differences in the authorship or primary purpose of each document may also contribute to observed inconsistencies. National Guidelines are technical documents that provide standardized protocols for HIV service delivery, National Strategic Plans describe a country's overarching strategy for addressing the HIV epidemic and COPs focus on PEPFAR‐aligned activities that are typically used for planning and implementation by national and bilateral agencies. While different target audiences will shape the details of PrEP implementation outlined within each document, ensuring alignment on core elements of PrEP implementation—for example, whom to prioritize for PrEP—is essential for streamlined and efficient implementation [29, 30].

Our results provide new insights into the scope of PrEP prioritization across the continent. These results, however, should be interpreted within the context of some limitations. First, by focusing on countries with PEPFAR‐supported PrEP programmes, our results disproportionately reflect PEPFAR priorities. Second, we only reviewed National HIV Treatment and Prevention Guidelines, National Strategic Plans and COPs published before July 2023. Other policy documents—for example, PrEP implementation plans—may have included additional details not summarized in this manuscript. Additionally, by reviewing only documents published before July 2023, our results describe national guidance for daily oral PrEP and do not reflect policies shaping access to new PrEP modalities. Third, our results reflect how countries described populations prioritized for PrEP in national guidance. Differences in the description of the same population group across documents or countries may have resulted in misclassification (e.g. high‐risk men and men who pay for sex). Finally, our results summarize the content of national policies rather than their implementation. As PrEP access and coverage may differ from the guidance outlined in these documents, future research is needed to better understand the extent to which national policies are implemented. This is especially important in contexts where key population groups are both prioritized for PrEP and criminalized.

## 5 CONCLUSIONS

Ending AIDS as a public health threat by 2030 requires radical reductions in HIV incidence across Africa. Enhancing PrEP uptake among individuals at risk of acquiring HIV is critical for achieving these reductions. Our review highlighted that while national PrEP policies in Africa are largely aligned with global guidance on PrEP offer, they often did not reflect the broader contextual and behavioural attributes of HIV risk, limiting populations with access to PrEP. Adapting policies to the local epidemiological context is critical for strengthening PrEP implementation and bringing global goals within reach.

## AUTHORS’ CONTRIBUTIONS

NER and CNM conceptualized the overall study. CNM collected all source documents. LAG, CNM and JW abstracted and analysed the data. LAG and CNM developed tables and figures and drafted the manuscript. All authors reviewed, edited, and revised the manuscript and approved the final submission.

## FUNDING

This study was funded by the National Institutes of Mental Health (NIMH) through awards R21‐MH125705 and R21‐MH134750. Additional investigator, trainee and administrative support was provided by the National Institute of Allergy and Infectious Diseases (NIAID) through awards T32‐AI007001, P30‐AI050410 and K24‐AI120796.

## Supporting information




Appendix

## Data Availability

The data that support the findings of this study were derived from the resources listed in the Supplementary File submitted with this manuscript. The abstracted data file is available from the corresponding author upon reasonable request.
