# Supplementary material for: Characterizing populations prioritized for PrEP in 19 African countries: a review of national guidance
Source: J Int AIDS Soc. 2025 Jan 5;28(1):e26407. doi: 10.1002/jia2.26407 (PMC11702491; doi:10.1002/jia2.26407)
Supplement: Supplementary file 1 — Appendix [file JIA2-28-e26407-s001.docx]

Characterizing populations prioritized for PrEP in 19 African countries: A review of national guidance

PrEP policy documents: References

Republique du Burundi Ministere de la Sante Publique et de la Lutte Contre le SIDA. Directives nationales pour la prevention et le traitement du VIH au Burundi. [Internet] Programme National de Lutte Contre le VIH/SIDA et les IST; 2020 [cited 2023 July 1]. Available from: <https://www.differentiatedservicedelivery.org/wp-content/uploads/burundi-2020.10-directives_nationales_tarv-2020-vf-prefacee_-09-octobre-2020.pdf>

U.S. President’s Emergency Plan for AIDS Relief (PEPFAR) Burundi. Burundi country operational plan (COP) 2022 strategic direction summary. [Internet] Burundi: PEPFAR Burundi; 2022 [cited 2023 July 1]. Available from: <https://www.prepwatch.org/wp-content/uploads/2022/11/Burundi-COP-2022.pdf>

Ministry of Health Ethiopia. National guidelines for comprehensive HIV prevention, care and treatment: Pocket guide. [Internet] MoH; 2022 [cited 2023 March 10]. Available from: <https://www.differentiatedservicedelivery.org/wp-content/uploads/POCKET-GUIDE-compressed-1.pdf>

Ministry of Health Ethiopia. HIV/AIDS National strategic plan for Ethiopia 2021 – 2025. [Internet] Federal HIV/AIDS Prevention and Control Office (FHAPCO); n.d [cited 2023 March 10]. Available from: <https://www.prepwatch.org/wp-content/uploads/2022/07/Ethiopia-HIVAIDS-National-Strategic-Plan-2021-25.pdf>

U.S. President’s Emergency Plan for AIDS Relief (PEPFAR) Ethiopia. Ethiopia country operational plan (COP) 2022 strategic direction summary. [Internet] Ethiopia: PEPFAR Ethiopia; 2022 [cited 2023 March 10]. Available from: <https://www.prepwatch.org/wp-content/uploads/2022/11/Ethiopia-COP-2022.pdf>

Ministry of Health, National AIDS & STI Control Program. Kenya HIV prevention and treatment guidelines, 2022 edition. [Internet] Nairobi, Kenya: NASCOP; 2022 [cited 2023 February 2]. Available from: <https://www.differentiatedservicedelivery.org/wp-content/uploads/Kenya-ARV-Guidelines-2022-Final-1.pdf>

National AIDS Control Council, Ministry of Health. Kenya AIDS strategic framework II 2020/21-2024/25: sustain gains, bridge gaps and accelerate progress. [Internet] MoH; n.d. [cited 2023 February 2]. Available from: <https://www.prepwatch.org/wp-content/uploads/2021/02/KenyaStrategicFramework_2021-2024.pdf>

U.S. President’s Emergency Plan for AIDS Relief (PEPFAR) Kenya. Kenya country operational plan 2022 strategic direction summary. [Internet] Kenya: PEPFAR Kenya; 2022 [cited 2023 February 2]. Available from: <https://www.prepwatch.org/wp-content/uploads/2022/11/Kenya-COP-2022.pdf>

Rwanda Biomedical Centre, Republic of Rwanda. Ministry of Health (MoH). National guidelines for prevention and management of HIV, edition 2020. [Internet] MoH; 2020 [cited 2023 February 10]. Available from: <https://www.prepwatch.org/wp-content/uploads/2022/08/Rwanda-National-HIV-Guidelines-for-Prevention-Management-of-HIV-2020.pdf>

Republic of Rwanda. Ministry of Health (MOH), Rwanda Biomedical Centre. Rwanda HIV and AIDS national strategic plan 2013-2018 extension 2018-2020*.* [Internet] MOH; 2018 2020 [cited 2023 February 10]. Available from: <https://rbc.gov.rw/fileadmin/user_upload/stra2019/strategie2019/Rwanda%20Strategic%20Plan%20for%20HIV%20Extended%20to%202020.pdf>

U.S. President’s Emergency Plan for AIDS Relief (PEPFAR) Rwanda. Rwanda country operational plan (COP/ROP) 2022 strategic direction summary. [Internet] Rwanda: PEPFAR Rwanda; 2022 [cited 2023 February 10]. Available from: <https://www.prepwatch.org/wp-content/uploads/2022/11/Rwanda-COP-2022.pdf>

United Republic of Tanzania. National guidelines for the management of HIV and AIDS*.* 7^th^ ed. [Internet] National AIDS Control Program; 2019 [cited 2023 February 10]. Available from: <https://differentiatedservicedelivery.org/Portals/0/adam/Content/NqQGryocrU2RTj58iR37uA/File/NATIONAL_GUIDELINES_FOR_THE_MANAGEMENT_OF_HIV_AND_AIDS_2019.pdf>

National AIDS Control Programme (NACP) of the United Republic of Tanzania. Health sector HIV and AIDS strategic plan (HSHSP IV) 2017-2022*.* [Internet] NACP; 2017. [cited 2023 February 15]. Available from: <http://library.tacaids.go.tz/bitstream/handle/123456789/128/HEALTH%20SECTOR%20HIV%20AND%20AIDS%20STRATEGIC%20PLAN%202018%20-%202022.pdf>

U.S. President’s Emergency Plan for AIDS Relief (PEPFAR) Tanzania. Tanzania country operational plan COP 2022 strategic direction summary. [Internet] Tanzania: PEPFAR Tanzania; 2022 [cited 2023 February 15]. Available from: <https://www.prepwatch.org/wp-content/uploads/2022/11/Tanzania-COP-2022.pdf>

Republic of Uganda, Ministry of Health. Consolidated guidelines for prevention and treatment of HIV and AIDS in Uganda. [Internet] MoH; 2020 [cited 2023 February 20]. Available from: <https://www.differentiatedservicedelivery.org/wp-content/uploads/Uganda_Consolidated-HIV-and-AIDS-Guidelines-2020-June-30th1.pdf>

Uganda AIDS Commission. The national HIV and AIDS strategic plan 2020/21 – 2024/25. [Internet] Uganda AIDS Commission; 2020 [cited 2023 February 20]. Available from: <https://uac.go.ug/index.php?option=com_content&view=article&id=24:hiv-prevention-1123&catid=8&Itemid=101>

U.S. President’s Emergency Plan for AIDS Relief (PEPFAR) Uganda. Uganda country operational plan (COP) 2022 strategic direction summary. [Internet] Uganda: PEPFAR Uganda; 2022 [cited 2023 February 20]. Available from: <https://www.prepwatch.org/wp-content/uploads/2022/11/Uganda-COP-2022.pdf>

Botswana Ministry of Health. Handbook of the Botswana 2016 Integrated HIV Clinical Care Guidelines. [Internet] MoH; 2016 [cited 2023 February 2]. Available from: <https://www.moh.gov.bw/Publications/Handbook_HIV_treatment_guidelines.pdf>

Republic of Botswana. National AIDS and Health Promotion Agency. The third Botswana national strategic framework for HIV and AIDS 2019-2023*.* 2019.

U.S. President’s Emergency Plan for AIDS Relief (PEPFAR) Botswana. BOTSWANA country operational plan (COP/ROP) 2022 strategic direction summary. [Internet] Botswana: PEPFAR Botswana; 2022 [cited 2023 February 3]. Available from: <https://www.prepwatch.org/wp-content/uploads/2022/11/Botswana-COP-2022.pdf>

Kingdom of Eswatini Ministry of Health. 2022 Eswatini Integrated HIV Management Guidelines. [Internet] MoH; 2022 [cited 2023 February 25]. Available from: <https://www.differentiatedservicedelivery.org/wp-content/uploads/Final-draft-for-printing_pdf.pdf>

Eswatini National Emergency Response Council for HIV/AIDS (NERCHA). The national multisectoral HIV and AIDS strategic framework 2018-2023*.* [Internet] NERCHA; 2018 [cited 2023 February 25]. Available from: <https://hivpreventioncoalition.unaids.org/wp-content/uploads/2019/06/Eswatini_NSF-2018-2023_final.pdf>

U.S. President’s Emergency Plan for AIDS Relief (PEPFAR) Eswatini. Eswatini country operational plan (COP) 2022 strategic direction summary. [Internet] Eswatini: PEPFAR Eswatini; 2022 [cited 2023 February 25]. Available from: <https://www.prepwatch.org/wp-content/uploads/2022/11/Eswatini-COP-2022.pdf>

Government of Lesotho. Ministry of Health (MOH). National guidelines on the Uue of antiretroviral therapy for HIV prevention and treatment*.* 6^th^ ed. [Internet] MOH; 2022 [cited 2023 January 28]. Available from: <https://www.differentiatedservicedelivery.org/wp-content/uploads/Final-Interactive-Lesotho-HIV-Prevention-Treatment-and-Care-guideline-2022.pdf>

Government of Lesotho. National AIDS Commission Secretariat. National HIV and AIDS strategic plan (NHASP) 2018/19- 2022/23*.* [Internet] National AIDS Commission Secretariat; 2018 [cited 2023 January 28]. Available from: <http://nac.org.ls/wp-content/uploads/2019/06/Lesotho-HIV-Strategic-Plan-2018-2023.pdf>

U.S. President’s Emergency Plan for AIDS Relief (PEPFAR) Lesotho. Lesotho country operational plan COP 2022 strategic direction summary. [Internet] Lesotho: PEPFAR Lesotho; 2022. [cited 2023 January 28]. Available from: <https://www.prepwatch.org/wp-content/uploads/2022/11/Lesotho-COP-2022.pdf>

Department of HIV and AIDS, Ministry of Health Malawi. National guidelines for the provision of oral pre-exposure prophylaxis for individuals at substantial risk of HIV in Malawi. [Internet] MOH; 2020 [cited 2023 March 14]. Available from: <https://dms.hiv.health.gov.mw/link/1qorf9bt>

National AIDS Commission (NAC) Malawi. Malawi national strategic plan for HIV and AIDS 2020 – 2025. [Internet] NAC; 2020 [cited 2023 March 14]. Available from: <https://www.prepwatch.org/wp-content/uploads/2022/07/National-Strategic-Plan-for-HIV-and-AIDS-2020-25-Final.pdf>

U.S. President’s Emergency Plan for AIDS Relief (PEPFAR) Malawi. Malawi country operational plan 2022 strategic direction summary. [Internet] Malawi: PEPFAR Malawi; 2022 [cited 2023 March 14]. Available from: <https://www.prepwatch.org/wp-content/uploads/2022/11/Malawi-COP-2022.pdf>

República de Moçambique. Guião de oferta da profilaxia pré-exposição ao HIV. [Internet] Ministério da Saúde; 2021 [cited 2023 April 2]. Available from: <https://www.prepwatch.org/wp-content/uploads/2022/07/Guide-to-Delivering-HIV-Pre-Exposure-Prophylaxis-2021.pdf>

Conselho Nacional de Combate ao HIV e SIDA. Plano estratégico nacional de combate ao HIV e SIDA (PEN V), 2021-2025. [Internet] Mozambique: 2021. [cited 2023 April 3]. Available from: <https://www.prepwatch.org/wp-content/uploads/2022/07/National-Strategic-Plan-of-Response-to-HIV-and-AIDS-NSP-V-2021-25.pdf>

U.S. President’s Emergency Plan for AIDS Relief (PEPFAR) Mozambique. Country operational plan (COP 2022) strategic direction summary PEPFAR Mozambique. [Internet] Mozambique: PEPFAR Mozambique; 2022 [cited 2023 April 4]. Available from: <https://www.prepwatch.org/wp-content/uploads/2022/11/Mozambique-COP-2022.pdf>

Republic of Namibia, Ministry of Health and Social Services. National guidelines for antiretroviral therapy: pocket guide edition, 2021. [Internet] 2021 [cited 2023 March 15]. Available from: <https://www.mhss.gov.na/documents/146502/1041983/2021+Clinical+Guidelines_Mobile.pdf/f0332fd1-df45-526b-ac91-7f0d51f292ad?t=1657529069248>

Republic of Namibia. Ministry of Health and Social Services (MOHSS). Directorate of Special Programmes. National strategic framework for HIV and AIDS response in Namibia 2017/18 to 2021/22*.* [Internet] MOHSS; 2017 [cited 2023 March 15]. Available from: <https://hivpreventioncoalition.unaids.org/wp-content/uploads/2018/05/NSF-Final-1.pdf>

U.S. President’s Emergency Plan for AIDS Relief (PEPFAR) Namibia. Namibia country operational plan 2022 strategic direction summary. [Internet] Namibia: PEPFAR Namibia; 2022 [cited 2023 March 15]. Available from: <https://www.prepwatch.org/wp-content/uploads/2022/11/Namibia-COP-2022.pdf>

Republic of South Africa, Department of Health (DOH). Guidelines for the provision of pre-exposure prophylaxis (PrEP) to persons at substantial risk of HIV infection*.* [Internet] South Africa: DOH; 2020 [cited 2023 February 15]. Available from: <https://www.prepwatch.org/wp-content/uploads/2020/07/South-Africa-PrEP-Guidelines_Jan2020.pdf>

South African National AIDS Council (SANAC). National strategic plan for HIV, TB, STIs, 2023-2028. [Internet] South Africa: SANAC; 2023. [cited 2023 July 24]. Available from: <https://www.differentiatedservicedelivery.org/wp-content/uploads/NSP-HIV-TB-STIs-2023-2028-MARCH20_23-PRINT2.pdf>

U.S. President’s Emergency Plan for AIDS Relief (PEPFAR) South Africa*.* Country operational plan PEPFAR South Africa 2022 strategic direction summary. [Internet] South Africa: PEPFAR South Africa; 2022 [cited 2023 February 18]. Available from: <https://www.prepwatch.org/wp-content/uploads/2022/11/South-Africa-COP-2022.pdf>

Republic of Zambia. Ministry of Health (MOH). Directorate of Clinical Care and Diagnostic Services. Zambia consolidated guidelines for treatment and prevention of HIV infection*.* [Internet] MOH; 2022 [cited 2023 April 20]. Available from: <https://www.differentiatedservicedelivery.org/wp-content/uploads/August-2022-Zambia-Consolidated-Guidelines.pdf>

National HIV/AIDS/STI/TB Council (NAC) Zambia. National HIV&AIDS strategic framework 2017-2021*.* [Internet] NAC; 2017 [cited 2023 April 20]. Available from:  <https://www.unicef.org/zambia/media/1031/file/Zambia-national-AIDS-strategic-framework-2017-2021.pdf>

U.S. President’s Emergency Plan for AIDS Relief (PEPFAR) Zambia. Zambia country operational plan (COP) 2022 strategic direction summary (SDS). [Internet] Zambia: PEPFAR Zambia;

2022 [cited 2023 April 20]. Available from: <https://www.prepwatch.org/wp-content/uploads/2022/11/Zambia-COP-2022.pdf>

Zimbabwe. National Medicines and Therapeutics Policy Advisory Committee (NMTPAC), Ministry of Health and Child Care (MOHCC). AIDS and TB Directorate. Guidelines for antiretroviral therapy for the prevention and treatment of HIV in Zimbabwe. [Internet] NMTPAC/MOHCC; 2016 [cited 2023 May 4]. Available from: <https://depts.washington.edu/edgh/zw/vl/project-resources/ZIM_ART_Guidelines_2016_-_review_final.pdf>

National AIDS Council of Zimbabwe. Zimbabwe national HIV and AIDS strategic plan 2021 – 2025. [Internet] Ministry of Health and Child Care; 2020 [cited 2023 May 4]. Available from: <https://www.prepwatch.org/wp-content/uploads/2023/03/ZIMBABWE-NATIONAL-HIV-STATEGIC-PLAN_2021-2025-1.pdf>

U.S. President’s Emergency Plan for AIDS Relief (PEPFAR) Zimbabwe. Zimbabwe country operational plan (COP) 2022 strategic direction summary (SDS). [Internet] Zimbabwe: PEPFAR Zimbabwe; 2022 [cited 2023 May 4]. Available from: <https://www.prepwatch.org/wp-content/uploads/2022/11/Zimbabwe-COP-2022.pdf>

Cameroon Ministry of Public Health. Directives nationales sur la prise en charge du VIH. [Internet] 2021 [cited 2023 April 20]. Available from: <https://www.differentiatedservicedelivery.org/wp-content/uploads/Directives_version-finale-05-aout-2021_Cameroon.pdf>

National AIDS Control Committee. Cameroon national strategic plan for fight against HIV/AIDS and STIs 2021-2023. [Internet] Ministry of Public Health; n.d. 2021 [cited 2023 April 20]. Available from: <https://www.prepwatch.org/wp-content/uploads/2022/10/Cameroon-National-Strategic-Plan-For-fight-against-HIVAIDS-and-STIs-2021-2023.pdf>

U.S. President’s Emergency Plan for AIDS Relief (PEPFAR) Cameroon. Cameroon country operational plan 2022 strategic direction summary. [Internet] Cameroon: PEPFAR Cameroon; 2022 2021 [cited 2023 April 20]. Available from: <https://www.prepwatch.org/wp-content/uploads/2022/11/Cameroon-COP-2022.pdf>

Programme National de Lutte Contre Le SIDA, République de Côte d’Ivoire. Plan stratégique national de lutte contre le VIH, le SIDA, et les infections sexuellement transmissibles 2021-2025. [Internet] 2020 2021 [cited 2023 April 25]. Available from: <https://www.pnlsci.com/wp-content/uploads/2022/06/psn-2021-2025-150620-table-de-matiere-revue-liste-de-presenence-01042022.pdf>

U.S. President’s Emergency Plan for AIDS Relief (PEPFAR) Cote d’Ivoire. Cote d’Ivoire country operational plan (COP/ROP) 2022 strategic direction summary. [Internet] Cote d’Ivoire: PEPFAR Cote d’Ivoire; 2022 2021 [cited 2023 April 25]. Available from: <https://www.prepwatch.org/wp-content/uploads/2022/11/Cote-dIvoire-COP-2022.pdf>

République Democratique du Congo. Programme National de Lutte Contre le Sida et les IST (PNLS). Guide de prise en charge intégrée du VIH en République Democratique du Congo*.* [Internet] PNLS; 2016 2021 [cited 2023 February 20]. Available from: <https://www.prepwatch.org/wp-content/uploads/2022/03/DRC-National-PrEP-Guideliens-2019.pdf>

Programme National Multisectoriel de Lutte contre le Sida (PNMLS) République Democratique du Congo. Plan stratégique national de la riposte au VIH/SIDA 2018-2021*.* [Internet] PNMLS; 2018 [cited 2023 February 20]. Available from: <https://www.ilo.org/dyn/natlex/docs/ELECTRONIC/111792/139484/F-1279925406/COD-111792.pdf>

U.S. President’s Emergency Plan for AIDS Relief (PEPFAR) Democratic Republic of Congo. Democratic Republic of the Congo country operational plan (COP) 2022 strategic direction summary. [Internet] Democratic Republic of the Congo: PEPFAR Democratic Republic of the Congo; 2022 [cited 2023 February 20]. Available from: <https://www.prepwatch.org/wp-content/uploads/2022/11/DRC-COP-2022.pdf>

National AIDS and STIs Control Programme, Federal Ministry of Health Nigeria. National guidelines for HIV prevention, treatment and care. [Internet] FMOH; 2020 [cited 2023 March 7]. Available from: <https://www.prepwatch.org/wp-content/uploads/2022/06/Nigeria-Guidelines-for-HIV-Prevention-Treatment-and-Care.pdf>

Federal Republic of Nigeria. National Agency for the Control of AIDS (NACA). National HIV and AIDS strategic plan 2017-2021*.* [Internet] NACA; 2017 [cited 2023 March 7]. Available from: <https://naca.gov.ng/wp-content/uploads/2018/05/National-HIV-and-AIDS-Strategic-Plan-FINAL1.pdf>

U.S. President’s Emergency Plan for AIDS Relief (PEPFAR) Nigeria. Nigeria country operational plan (COP) 2022 strategic direction summary. [Internet] Nigeria: PEPFAR Nigeria; 2022 [cited 2023 March 7]. Available from: <https://www.prepwatch.org/wp-content/uploads/2022/11/Nigeria-COP-2022.pdf>
